# Mapping the protein–protein interactome in the tumor immune microenvironment

**DOI:** 10.1093/abt/tbad026

**Published:** 2023-11-14

**Authors:** Rui Peng, Mi Deng

**Affiliations:** Peking University International Cancer Institute, Health Science Center, Peking University, Beijing 100191, PR China; School of Basic Medical Sciences, Health Science Center, Peking University, Beijing 100191, PR China; Peking University International Cancer Institute, Health Science Center, Peking University, Beijing 100191, PR China; School of Basic Medical Sciences, Health Science Center, Peking University, Beijing 100191, PR China; Peking University Cancer Hospital and Institute, Peking University, Beijing 100142, PR China

**Keywords:** protein–protein interaction, target discovery, interactome, tumor immune microenvironment, immuno-oncology

## Abstract

The cell-to-cell communication primarily occurs through cell-surface and secreted proteins, which form a sophisticated network that coordinates systemic immune function. Uncovering these protein–protein interactions (PPIs) is indispensable for understanding the molecular mechanism and elucidating immune system aberrances under diseases. Traditional biological studies typically focus on a limited number of PPI pairs due to the relative low throughput of commonly used techniques. Encouragingly, classical methods have advanced, and many new systems tailored for large-scale protein–protein screening have been developed and successfully utilized. These high-throughput PPI investigation techniques have already made considerable achievements in mapping the immune cell interactome, enriching PPI databases and analysis tools, and discovering therapeutic targets for cancer and other diseases, which will definitely bring unprecedented insight into this field.

## Teaser

Statement of Significance: Conclusion of various systems capable of high-throughput PPI investigation emerged in recent years. Preliminary analysis of the causes of deviations in different methods.

## INTRODUCTION

The immune system is a complex and dynamic network of cells that communicate with each other through protein–protein interactions (PPIs) to fight against infection and disease. PPIs are also essential for immune cell differentiation and development, executing immune functions and maintaining tissue homeostasis throughout the body. Cell-to-cell communication in the immune system is primarily mediated by the receptor–ligand associations on the cell surface. These receptors and ligands are potential therapeutic targets for various diseases, as they play pivotal roles in regulating immune response. Extensive research has been conducted on immune checkpoint molecules, such as programmed cell death protein 1 (PD-1), cytotoxic T-lymphocyte associated protein 4 (CTLA-4) and programmed cell death ligand 1 (PD-L1). These proteins are validated drug targets for the treatment of cancer, as they can be used to normalize the immune surveillance functions. Monoclonal antibodies targeting B-cell activation receptors and ligands, such as clusters of differentiation 20 (CD20), have also been widely used as first-line drugs for multiple cancers and autoimmune diseases. Overall, understanding the mechanisms of cell-to-cell communication in the immune system and identifying potential targets for therapeutic intervention is a promising avenue for the treatment of a wide range of diseases.

Traditionally, researchers have focused on a limited number of interacting pairs to study their mechanisms of action and functions. However, the advent of high-throughput, high-content and multi-omics technologies has enabled the study of numerous receptor and ligand interactions in batches. This capability has made it possible to analyze complex interactions and networks which were previously difficult or impractical to study. These advances have paved the way for the systematic mapping of the immune cell interactome and the identification of valuable therapeutic targets on a large scale.

## HIGH-THROUGHPUT METHODS FOR PPI IDENTIFICATION

A variety of methods and platforms have been developed for large-scale exploration of PPIs, many of which are suitable for studying the receptor and ligand interactions of immune cells. Classical methods to investigate PPIs include yeast two-hybrid (Y2H), affinity purification-mass spectrometry (AP-MS), co-immunoprecipitation (Co-IP), mammalian protein–protein interaction trap (MAPPIT), bimolecular fluorescence complementation (BiFC), surface plasmon resonance (SPR), bioluminescence/F$\ddot{\text{o}}$rster (fluorescence) resonance energy transfer (BRET/FRET), proximity ligation assay (PLA), Glutathione S-Transferase (GST) pull-down assay, Bio-Layer Interferometry (BLI), Phage-Display and avidity-based extracellular interaction screening (AVEXIS) [1]. These methods span a wide range of techniques from molecular biology, genetics, to biophysics, and vary in sensitivity, specificity and scalability. Their advantages and disadvantages have been well documented in the literature (Table 1).

**Table 1:** Classical methods for protein–protein interaction investigation

Methods	Principle	Advantages	Disadvantages
Co-IP LUMIER	Use antibodies to precipitate proteins and their ligands together	• Easy to perform • Can be used in HT screening • Can be used in different cell lines	• Indirect interactions can also be detected • Difficult for weak and transient PPI • Carefully control to normalize for differences in transfection efficiency and expression
Y2H MYTH MaMTH	Physical separation of two functional moieties of a transcription factor which is fused to candidate interacting proteins	• Simple, well established and low cost • Can be used in HT screening • Needless of cell lysis	• Lack of necessary posttranslational modifications • Non-specific interactions due to overexpression • Prevent spatial or temporal analysis of PPIs
FRET BRET	Non-radiative transfer of energy from an excited donor fluorophore to a nearby acceptor molecule	• Able to monitor instantaneous, real-time PPIs • Can be used in the live cells and allows detection of interaction sites • Monitor complex interaction dynamics	• Need to consider the functionality of fusion proteins • Need close spatial proximity for strong readout • Is not easily scalable to HT screening
BiFC	Division of a fluorescent protein (e.g., YFP) into two distinct non-fluorescent fragments, which are fused to “bait” and “prey” proteins^#^	• Able to monitor instantaneous, real-time PPIs • Can be used in the live cells and allows detection of interaction sites • Monitor complex interaction dynamics	• Unsuitable for PPI dynamics or real-time changes • False-positive fluorescent signals • Need to consider the functionality of fusion proteins
MAPPIT KISS	A bait protein is fused to a cytokine receptor deficient in binding to STAT3, prey proteins are fused to STAT3 recruitment sites	• Easy to perform • Can be used in HT screening • Effective for use in small-molecule screening approaches	• Requires that PPIs occur in the cytoplasmic submembrane region • Large size of the bait tag may also block certain PPI • Unsuitable for spatial or temporal analysis
SPR	Bait protein is coated on a sensor chip and the total reflection resonance angle would be changed when prey protein binds to the bait under flow	• Highly sensitive • No need to tag detected protein • Monitor interaction dynamics and access multi dynamics parameters (*K*_d_, *K*_a_)	• Very sensitive to motion and temperature • Require purified protein • Relative low throughput
AP-MS BioID-MS	Use co-immunoprecipitation method, proximity-dependent biotin identification method etc. to enrich prey proteins which can bind to the bait protein and use mass spectrometry to decipher the enriched component	• Library independent with genome-wide throughput to a specific protein	• Difficult data analysis • AP-MS unsuited for weak and transient PPI
PLA	Both bait and prey proteins are targeted with primary antibody, which is targeted by secondary antibodies with DNA fragments. When proteins are in proximity, the DNA fragments together with subsequently added oligonucleotide fragments can form a circular DNA that can be amplified and detected by fluorophore-labeled complementary oligonucleotide probes	• Localize PPIs • Suitable for weak and transient interaction	• Relative low throughput
GST Pull-Down Assay	Bait proteins are fused with GST gene and purified on glutathione-agarose beads. Prey proteins are incubated together with these beads and resolved by SDS-PAGE western blotting, autoradiography or staining	• Able to detect low-abundant protein interaction	• Need to fuse with GST tag
BLI	Bait proteins are coated on a biosensor tip. The interference patterns of white light reflected from the surface of the biosensor tip are monitored in real time, which will be changed when bait proteins bind to prey proteins	• Provides real-time interaction dynamics • Highly sensitive • Label-free	• Can only monitor a few interactions simultaneously • Expensive • Poor reproducibility
Phage-Display	Bait proteins are genetically fused with the coat proteins of bacteriophage, leading to the production of phage particles displaying bait proteins on their surface. After binding with prey proteins, bacteriophages with interest can be isolated	• Can be used in HT screening • Detect in a biological context	• Proteins may change their structure and functionality when displayed on bacteriophage • Difficult to display long peptides

^#^“bait” refers to the query receptor and “prey” refers to the query ligand.

**Figure 1:**
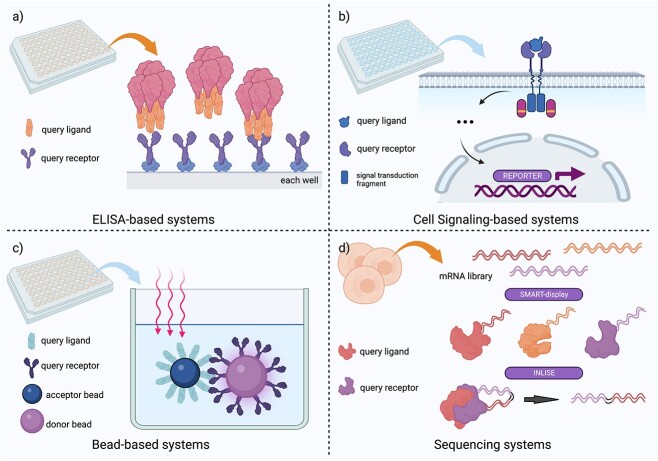
Newly advanced systems for high-throughput protein–protein interaction screening. In ELISA-based systems, the query receptors will be captured on a coated plate, while the tagged query ligands which is linked to chromogenic reaction enzyme will be added to the plate in the form of an ELISA test. In cell signaling-based systems, chimeric receptors containing both the extracellular domain of query proteins and the intracellular fragments of signal transduction molecules will be activated and induce a cell signal cascade when successfully bind to query ligand. In bead-based systems, protein pairs in query would either captured by donor beads or acceptor beads which can chemiluminate when the protein pairs interacted. In sequencing systems, cell would be lysed and the obtained mRNA library would be transformed into conjugate proteins with their mRNA barcode. When proteins interact, their mRNA barcode can form chimeric sequence which could be sequenced and deciphered. This figure was created with BioRender.com.

Researchers have made significant discoveries by choosing the appropriate PPI methods that are tailored to their specific goals. However, the challenges posed by low binding affinity and high background signal have significantly limited the study of extracellular protein interactions at larger scales and higher resolutions. Fortunately, recent advancements have yielded innovative platforms with well-defined workflows that can be easily used by researchers to address these challenges. These platforms either build upon classical PPI methods or pioneer entirely novel approaches (Fig. 1).

### Enzyme-linked immunosorbent assay-based methods

AVEXIS is a modified enzyme-linked immunosorbent assay (ELISA) method that is specifically designed to detect low affinity and transient protein–protein interactions. In this assay, both the “bait” and “prey” proteins are expressed and purified. The biotinylated “bait” proteins are immobilized on a streptavidin-coated plate, and the “prey” proteins are fused with the pentameric β-lactamase to allow avidity driven binding. The “prey” proteins are then added to the plate. If they bind to the “bait” proteins, the β-lactamase-catalyzed chemical reactions can be detected and quantified to measure the strength of the specific interactions. In 2022, Jarrod Shilts *et al*. improved the AVEXIS method, successfully enabling it for larger-scale screenings, and called this improved system the scalable arrayed multi-valent extracellular interaction screen (SAVEXIS) [2]. SAVEXIS automated the original AVEXIS assay and exploited multimerization around streptavidin for both “bait” and “prey” proteins. The new method can be used to screen larger numbers of interactions with greater sensitivity and reduced workload. Using this method, Jarrod Shilts *et al*. screened 630 different surface proteins of immune cells, which include almost all types of extracellular proteins found on immune cells, and generated a high-confidence surface protein interactome. Nadia Martinez-Martin *et al*. used another modified version of AVEXIS method to screen PPIs involving pathogen–host interactions [3]. In this assay, the “prey” proteins were connected to β-lactamase by a pentameric helical region derived from rat cartilage oligomeric matrix protein (COMP), resulting in the pentameric form of the “prey” proteins. The “bait” proteins were fused with the Fc portion of human IgG protein and captured on protein A-coated plates. The researchers constructed a library of about 1300 unique single-pass transmembrane receptors to screen for the long-sought receptor of human cytomegalovirus. They identified neuropilin-2 as its receptor. Using the same platform, they screened the PPIs of 445 immunoglobulin superfamily (IgSF) proteins and 1364 human receptor proteins, leading to the identification of an IgSF interactome with 472 novel interacting pairs that were undocumented previously. Another platform created by Wojtowicz *et al*. in 2020 combined the ELISA-based extracellular interactome assay (ECIA) with an automated pooled-prey strategy, known as apECIA. This platform has some distinct variations that allow large-scale screens [4]. In the apECIA assay, three “prey” proteins are pooled per well with one “bait” protein. The interactions are then deconvoluted after the screening. Other ELISA-based large-scale screening platforms can be found in different works. It has been shown that ELISA-based binding assays have a low false-positive rate. Although those methods vary in details, most of them introduce automated workflow and multimerized query proteins to achieve high throughput and high sensitivity. These ELISA-based methods are remarkably fit for the investigation of PPIs of cell-surface and secreted proteins (Fig. 1a).

### Cell signaling transduction-based methods

Another frequently used method for large-scale PPI screening involves cell signal transduction. In this approach, chimeric receptors are engineered by fusing the extracellular domain of query proteins with the intracellular fragments of signal transduction molecules. When ligands bind to the chimeric receptors, the receptors are activated, initiating signal transduction cascades that eventually lead to the activation of a reporter gene, such as the green fluorescent protein (GFP) (Fig. 1b). MAPPIT is a classical cell signaling transduction-based method that exploits the Janus kinase/signal transducers and activators of transcription (JAK/STAT) signaling pathway to detect PPIs [5]. Other signaling pathways have also been used to capture receptor and ligand interactions. The performance of MAPPIT has been continuously optimized, and it is now a widely used method for investigating PPIs in living cells. Wang *et al*. developed a high-throughput functional screening system called T cell activity array (TCAA) in 2019 to screen cell surface proteins that could modulate T-cell activities [6]. In the TCAA system, query proteins were expressed on the cell surface of the artificial antigen-presenting cell line (aAPC). The aAPCs were then used to activate a Jurkat cell line containing the nuclear factor κB (NF-κB) and nuclear factor of activated T cells (NFAT) reporter gene. Any PPIs that modulate the T-cell activation can be identified for further study. They screened over 6500 human genes encoding more than 90% of transmembrane proteins in the human genome and identified sialic acid-binding Ig-like lectin 15 (Siglec-15) as a critical immune suppressor. In another study, Barrow *et al*. constructed chimeric receptors by fusing the extracellular and transmembrane domains of human proteins with the intracellular signaling domain of clusters of differentiation 3 zeta chain (CD3ζ) [7]. These chimeric receptors were expressed on a GFP reporter cell line that was responsive to the CD3ζ-driven Ca^2+^-NFAT signaling pathway activation when it was associated with activation ligands. With this system, the team screened a secretome protein library containing about 4000 human and mouse proteins, as well as ectodomains of some single-pass transmembrane proteins, to find the undetermined ligand for natural cytotoxicity triggering receptor 2 (NCR2). They finally discovered that platelet derived growth factor D was the ligand for NCR2.

### Integrated omics platforms

Some other emerging large-scale PPI research methods have shown great promise. For example, Zhong *et al*. creatively combined the nucleotide sequencing techniques with PPI investigation in their newly developed system known as protein–protein interaction sequencing (PROPER-seq) [8]. In the first step, proteins were conjugated with a barcode mRNA using a modified mRNA-display method called SMART-display. This transformed the input cells into a barcoded protein library. When the protein complex formed, the associated barcode mRNAs were also brought into close proximity, which could then be ligated to produce a chimeric barcoded sequence. This new barcode sequence can be sequenced to reveal the identities of the interacting proteins (Fig. 1d). This method transformed the PPI investigation into a sequencing-based approach, enabling human genome-wide PPI screening and the identification of more than 200 000 previously uncharacterized human PPIs. Baryshev *et al*. advanced Y2H technique and developed the massively parallel protein–protein interaction measurement by sequencing (MP3-seq) systems [9]. They conjugated DNA barcodes with specific protein pairs and the interaction ability of these pairs can then be evaluated by sequencing and counting the occurrence of barcodes in the sample. This allowed them to identify PPIs that were enriched in the sample. MP3-seq was validated by its ability to detect the interactions between B-cell lymphoma 2 (Bcl-2) and its interacting partner. Martinez-Martin and her team also developed a bead-based PPI screening system called automated conditioned media alpha screen [10]. In this system, protein pairs of interest were captured by either donor or acceptor beads. These beads were chemiluminated upon the protein pairs interacting, providing a quantifiable signal that reflects the occurrence of protein interactions (Fig. 1c). Wang and colleagues also developed a genome-scale receptor array technology based on antibody binding and flow cytometry. This technology successfully identified fibrinogen-like protein 1 (FGL1) as the major inhibitory binding partner of the ligand of lymphocyte-activation gene 3 (LAG-3), out of approximately 5600 human transmembrane proteins and 1000 secreted proteins [11]. Another study utilized a cell-based genome-wide approach employing CRISPR activation to identify neurite outgrowth inhibitor (Nogo), one of the myelin-associated inhibitory proteins, as the ligand for adhesion G protein-coupled receptor B1 (ADGRB1) [12]. Oslund and his team advanced the proximity labeling technology through the introduction of photocatalytic cell tagging (PhoTag) for profiling cell–cell interactions [13]. By integrating PhoTag with multi-omics single-cell sequencing, they revealed the different interaction efficiency of specific T-cell subtypes with Raji PD-L1 B cells, and that these T-cell types possessed distinct transcriptional programs.

### In silico PPI prediction

The advancement of biotechnology and computer technologies has enabled the computational prediction of PPI. This has led to the development of a variety of in silico methods for large-scale PPI predictions, which leverage insights from both the experimental data and computational algorithms. There are several different types of techniques for PPI predictions (Table 2). Sequence-based methods, such as Bock and Gough [14], PPIevo [15] and VLASPD [16], predict PPIs by analyzing the amino acid sequences of proteins involved. Structure-based methods, such as PrePPI [17] and MEGADOCK [18], predict PPIs by analyzing the three-dimensional (3D) structure of proteins of interest.

**Table 2:** In silico PPI prediction methods

Methods	Tools	Advantages	Disadvantages
Sequence-based methods	Bock and Gough [14] PPIevo [15] VLASPD [16]	• All protein amino sequences are available, including those of unstable or intrinsically disordered proteins • Time-saving and easy to conduct	• Usually less accurate • False positives, overfitting and underfitting • May need additional information to improve performance • Difficult to decipher the interaction mechanism
Structure-based methods	PrePPI [17] MEGADOCK [18]	• More accurate • Consistent with biological processes • Model PPI at the atomic level and provide insights into the underlying interaction mechanisms	• Hard to apply to proteins lack 3D-structure information • Generally time consuming and requires expensive computation resources
Deep learning-based methods	SAE [19] DPPI [20] DNN-PPI [21] MaSIF [22]	• Higher accuracy • Can learn complex patterns from high-dimensional data	• Deep-learning models are hard to explain • Demanding for computation resources
Ortholog-based methods	Sheng-An Lee *et al*. [23]	• Easy to conduct	• Limited to predict proteins with ortholog and known ortholog PPIs • Unstable as ortholog proteins may different in protein interaction
Gene fusion-based methods	Enright *et al*. [24]	• Utilizes gene location information	• Limited in use • Less accurate, need additional information
Network topology-based methods	L3 [25] SFCN [26]	• Beneficial for investigating functional interaction network	• Difficult to predict the interactions between sparsely connected proteins

Sequence-based methods leverage the wealth information within protein sequences. These methods use the primary sequence and additional information such as physicochemical properties of amino acids and sequence similarity scores, as input to generate predictive models. Classical models such as support vector machines and random forests are often used to assess the likelihood of PPIs and classify them into interactive or non-interactive pairs. Sequence-based methods are generally less accurate than other methods, and can be prone to false positives, overfitting and underfitting. To improve the performance of sequence-based PPI prediction methods, it is often necessary to incorporate additional information, such as functional or evolutionary data. Furthermore, it can be challenging to decipher the interaction mechanism after prediction using these methods. Despite their limitations, sequence-based methods remain valuable in PPI prediction. This is because amino acid sequence information is available for all proteins, including those that lack structural information. Sequence-based methods are also more time-efficient and easier to implement than other methods. Therefore, they are a viable option for guiding experimental design or cases where high accuracy is not a strict requirement.

Structure-based methods that use the 3D structures of proteins to predict PPIs are more informative and consistent with biological processes than sequence-based methods. These methods model PPI at the atomic level and provide insights into the underlying interaction mechanism. Structure-based methods are diverse in the underlying theoretical principles, but some common approaches include template-based prediction techniques, which align the structural surfaces of the target PPI with known PPI interfaces; and domain-based techniques, which treat domains as the basic units of PPI and aim to predict the most likely interacting domains. Machine learning models are commonly used in structure-based PPI prediction methods because they can incorporate protein structural information to various extents, including local docking methods or global docking methods. Although structure-based methods tend to be more accurate than sequence-based methods, their applicability is constrained by the availability of protein structural data. Data for structure-based models are often limited, and the models are difficult to apply to proteins lacking 3D structural information. Besides, determining the 3D structure of a protein demands a specialized knowledge and skill-sets, and structure-based methods are generally time consuming and requires expensive computational resources.

In recent years, the rapid development of deep learning methodologies has introduced transformative tools for PPI prediction, such as SAE [19], DPPI [20], DNN-PPI [21] and MaSIF [22]. MaSIF, in particular, has demonstrated its capability in deciphering protein–protein interaction interface patterns. Deep learning methods can use either sequence or structural information to analyze protein interaction patterns based on existing PPI data. These methods are promising for PPI predictions because they can learn complex patterns from high-dimensional data, which is essential for capturing the complex features involved in PPIs. As we expected, these methods have already outperformed other competing methods and demonstrated their potential to fully decipher the PPI mechanism.

In addition to these methods, a variety of other methods have been developed for PPI prediction, including ortholog-based techniques, gene fusion-based approaches and network topology-based techniques. Ortholog-based techniques leverage the knowledge of conserved interactions in other species to predict the interacting networks of target proteins [23]. Gene fusion-based approaches propose that genes in close proximity are fused under selective pressure over the course of evolution and have greater tendency to interact with each other [24]. Network topology-based techniques view the protein interaction networks as an undirected graph and exploit the patterns of known interactions to identify new interactions [25, 26]. The development of these diverse methods has led to the growing prosperity in the field of PPI prediction.

## P‌PIS: A KEY TO UNLOCKING THE MYSTERIES OF TUMOR IMMUNITY

PPIs are essential for many biological processes, including immunity. The successful applications of large-scale PPI screenings have benefited a wide range of research, and they can be extended to countless other studies requiring PPI investigation in any field. Large-scale screening is the most effective and comprehensive way to discover the binding partners for functionally important orphan receptors, and it is also the most efficient approach to systematically map all types of interactomes. With the PPI data, it is possible to measure interactions between different cell types, understand the mechanisms of numerous biological processes and discover novel therapeutic targets with a reduced risk of side effects. This approach is remarkable for its ability to achieve comprehensive results on a broad scale within a short time frame.

**Figure 2:**
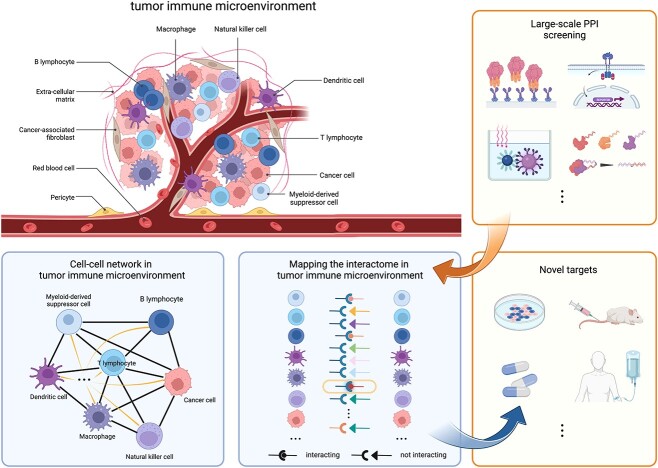
High-throughput PPI screening maps the protein–protein interactome in the tumor immune microenvironment. Cell-to-cell communication within the TIME is exceptionally active and dynamic, forming a complex network of PPIs on a large scale. High-throughput PPI screening maps the protein–protein interactome and identifies novel possible targets for the additional function assays. This figure was created with BioRender.com.

Large-scale screening is particularly valuable in the field of tumor immunity. The tumor immune microenvironment (TIME) is a specialized immune niche where infiltrating and resident immune cells, stromal cells, blood vessels and extracellular matrix surround tumor cells to form a complex and continuously evolving entity. Immune cells in the TIME are diverse in types and states, working in coordination to execute their unique functions or counteracting each other to inhibit and exhaust their capabilities. Cell-to-cell communication within the TIME is exceptionally active and dynamic, forming a complex network of PPIs on a large scale. These PPIs are the subject of intensive investigation, as they play a pivotal role in regulating tumor immunity. High-throughput techniques are crucial for revealing novel functional targets within this intricate network that could be used for therapeutic purposes (Fig. 2).

### Mapping the immune cell interactome

The most direct use of large-scale PPI research is to systematically map various interactomes, providing tremendous PPI information for further investigation. The complete immune physical wiring diagram has been accomplished, which systematically maps the network of immune cells formed by the interactions of leukocyte cell-surface proteins. In recent years, interactome maps for specific protein types, such as IgSF family proteins, single-pass transmembrane proteins and secreted proteins, have also been completed [2, 4, 27]. These interactome maps have identified numerous important interacting partners for some orphan receptors. For example, vascular endothelial growth factor receptor 3 (VEGFR3) and programmed cell death 1 ligand 2 (PD-L2) were discovered as the ligands of the orphan granulocyte receptor CEA cell adhesion molecule 4 (CEACAM4), which is involved in the regulation of immune cell trafficking and differentiation [2]. The immune checkpoint receptor V-domain Ig suppressor of T cell activation (VISTA) was deorphanized by discovering that the non-classical major histocompatibility complex (MHC) molecules human leukocyte antigen-E (HLA-E) and HLA-F are its endogenous non-tumor ligands [2]. The prospective orphan checkpoint inhibitor B7 homolog 3 protein (B7-H3), which is an attractive target for immunotherapy and has reached its phase II clinical trial, was also found to bind to the interleukin 20 receptor α (IL20RA) [10]. Two different large-scale PPI screenings independently identified poliovirus receptor (PVR) protein as the ligand for the previously orphan receptor killer cell immunoglobulin like receptor, two Ig domains and long cytoplasmic tail (5AKIR2DL5A) [4, 10].

### Novel therapeutic target identification by PPI screening

A good example of how large-scale PPI screening has promoted the identification of novel therapeutic targets is the discovery of fibrinogen-like protein 1 (FGL1) as a ligand of LAG-3. FGL1 is a promising target for new generation immunotherapy research [11]. LAG-3 was first discovered to interact with MHC II, and was thought to negatively regulate T-cell activation by competing with clusters of differentiation 4 (CD4). However, several studies have reported that LAG-3 can function independently of MHC II association. Chen and his team performed a large-scale PPI screening to investigate LAG-3 binding partners, and FGL1 was identified as its major functional ligand, providing a more comprehensive understanding of this field. FGL1 was identified as a tumor-associated factor many years ago. It was highly expressed in most solid tumors, including lung, prostate, melanoma, gastric and breast cancers [11, 28, 29]. FGL1 expression level is associated with epithelial-mesenchymal transition, tumor angiogenesis, proliferation and apoptosis, drug resistance and tumor immune escape [30]. The discovery of FGL1/LAG3 interaction has helped to partially clarify these processes. In addition, FGL1 and LAG3 are being investigated as potential therapeutic targets for patients who are unresponsive to anti-PD-1 and anti-PD-L1 therapies. High FGL1 level in the blood is significantly associated with poor prognosis in patients with non-small cell lung cancer (NSCLC) and melanoma who have been treated with anti-PD-1/PD-L1 [31] antibodies. Animal experiments have shown that anti-FGL1 or anti-LAG-3 therapies, either as a monotherapy or in combination with anti-PD-1/PD-L1 therapies, can have tumor suppressive effects. This suggests FGL1/LAG3 and PD-1/PD-L1 pathways can independently regulate T-cell activity and blocking both pathways can produce synergistic antitumor effects. Since the establishment of FGL1 as a high-affinity ligand of LAG-3 in 2019, the pharmaceutical industry has shown great enthusiasm in developing anti-FGL1 immunotherapy for cancer patients, especially NSCLC patients. Although the potential therapeutic efficacy of this interaction has only been identified in a preclinical stage, the substantial evidence gathered to date points to an exciting and promising prospect of FGL1 for immunotherapy. The realization of anti-FGL1 clinical application is truly worth waiting for.

Other renowned and promising immune checkpoint targets, such as T-cell immunoreceptor with immunoglobulin and ITIM domain (TIGIT) and T-cell immunoglobulin and mucin-domain containing-3 (TIM-3), have also benefited from large-scale PPI screening. These studies have enabled a much more comprehensive understanding of their interaction networks. Large-scale PPI screening systems have identified new interaction partners for several hotspot molecules, including carcinoembryonic antigen-related cell adhesion molecule 1 (CEACAM1) for PD-1, ephrin type-A receptor 3 (EPHA3), natural killer protein 30 (Nkp30) for PD-L1, reversion-inducing cysteine-rich protein with Kazal motifs (RECK), FcγRIIa for TIGIT, family with sequence similarity 200 member A (FAM200A), WW domain-binding protein 1 (WBP1) and protocadherin gamma subfamily B, 4 (PCDHGB4) for CTLA4. The findings have provided a comprehensive understanding of the interaction modes for these promising therapeutic targets, which has boosted the development of numerous drugs that have entered into clinical trials. The newly detected interaction between PD-L1 and EPHA3 is particularly interesting, as it may have implications for cancer therapy. An EPHA3 monoclonal antibody, KB004, had already entered clinical trials for patients with glioblastoma and advanced hematologic malignancies before this interaction was discovered [32, 33]. This discovery may improve the understanding of this therapeutic target and suggest a synergistic effect between PD-L1 inhibitor and EPHA3 antibody, which warrants further study.

### P‌PI-based prediction of cell–cell communication

The availability of interactomes has enabled the use of in silico methods to estimate the interplay among various cell types. This can shed light on many immunology mechanisms, such as the reciprocal influence between immune cells and cancer cells in the tumor microenvironment. Several major PPI interaction databases have been established to facilitate this endeavor (Table 3), including IntAct [34], BioGRID [35], STRING [36], IID [37], iRefWeb [38] and MatrixDB [39]. These databases collected a majority of the confirmed PPIs and are constantly being updated. Large-scale PPI screening significantly enriches these databases with comprehensive information. Various computational tools are available to evaluate the cell communication by utilizing the PPI data from these databases, alongside newly discovered interactomes and the expression matrix of query cell types (Table 4). Key tools include CellChat [40], CellPhoneDB [41], CellTalker [42], Connectome [43], ICELLNET [44], iTalk [45], NATMI [46], NicheNet [47] and SingleCellSignalR [48]. While these tools employ diverse algorithms, they collectively provide predictions of cell–cell communication based on the established PPI information. Although most predictions mainly focused on inferring interactions between two cell types, the recently introduced Scriabin tool has shown the potential to infer biological edges of cell–cell communication at single-cell resolution [49].

**Table 3:** Protein–protein interaction databases

Database	Latest update	Reference	Link
STRING	2023	The STRING database in 2023: protein–protein association networks and functional enrichment analyses for any sequenced genome of interest. *Nucleic Acids Res*	http://string-db.org
IntAct	2022	The IntAct database: efficient access to fine-grained molecular interaction data. *Nucleic Acids Res*	http://www.ebi.ac.uk/intact
IID	2021	IID 2021: towards context-specific protein interaction analyses by increased coverage, enhanced annotation and enrichment analysis. *Nucleic Acids Res*	http://ophid.utoronto.ca/iid
iRefWeb	2021	Navigating the global protein–protein interaction landscape using iRefWeb. *Methods Mol Biol*	http://wodaklab.org/iRefWeb/
BioGRID	2019	The BioGRID interaction database: 2019 update. *Nucleic Acids Res*	http://thebiogrid.org
MatrixDB	2019	MatrixDB: integration of new data with a focus on glycosaminoglycan interactions. *Nucleic Acids Res*	http://matrixdb.ibcp.fr/
InnateDB	2013	InnateDB: systems biology of innate immunity and beyond—recent updates and continuing curation. *Nucleic Acids Res*	http://www.innatedb.ca
MINT	2012	MINT, the molecular interaction database: 2012 update. *Nucleic Acids Res*	http://mint.bio.uniroma2.it/mint
DIP	2004	The Database of Interacting Proteins: 2004 update. *Nucleic Acids Res*	http://dip.doe-mbi.ucla.edu/dip

**Table 4:** Cell-to-cell communication analysis tools based on protein–protein interactions

Tools	Year	Reference	Platform
CellChat	2021	Inference and analysis of cell–cell communication using CellChat. *Nat Commun*	R
CellPhoneDB	2020	CellPhoneDB: inferring cell–cell communication from combined expression of multi-subunit ligand-receptor complexes. *Nat Protoc* 2020	Python
CellTalker	2020	Immune landscape of viral- and carcinogen-driven head and neck cancer. *Immunity*	R
Connectome	2022	Computation and visualization of cell-cell signaling topologies in single-cell systems data using Connectome. *Sci Rep*	R
ICELLNET	2021	Dissection of intercellular communication using the transcriptome-based framework ICELLNET. *Nat Commun*	R
iTalk	2019	Dissection of intercellular communication using the transcriptome-based framework ICELLNET. *Nat Commun*	R
NATMI	2020	Predicting cell-to-cell communication networks using NATMI. *Nat Commun*	Python
NicheNet	2020	NicheNet: modeling intercellular communication by linking ligands to target genes. *Nat Methods*	R
SingleCellSignalR	2020	SingleCellSignalR: inference of intercellular networks from single-cell transcriptomics. *Nucleic Acids Res*	R
Scriabin	2023	Comparative analysis of cell–cell communication at single-cell resolution. *Nat Biotechnol*	R

## THE VARIATION ENIGMA IN LARGE-SCALE PPI SCREENING

We have observed that the interaction landscape varies across different large-scale PPI screening systems. This is because different systems use different methods and criteria to detect interactions, and they may also be subject to different biases. As a result, the same interacting pairs may be detected in one system but not in another. The reasons for these discrepancies are complex and multifaceted, but they also indicate a lack of integration and standardization in this field. One factor that can contribute to variation in PPI screening is the nature of the protein itself. For instance, the interaction properties of membrane proteins can be influenced by the specific lipid environment in which they reside. Membrane proteins are typically found in the cell membrane, where they interact with other proteins and the surrounding lipids. When these proteins are isolated from the membrane, they may adopt a different conformation and lose their abilities to interact with other proteins. Also on the cell surface, they have binding partners. One example is CD47, which is a component of a big complex, which is missed in its purified form. Cell-based systems are more similar to real biological context than that of other methods to detect PPIs. However, they rely on signal transduction and reporting, which can result in detection sensitivity variations depending on the signal pathway used. The protein preparation protocol also matters since proteins are usually expressed and purified from cell lines. Different expression cell lines may cause certain modification variances such as glycosylation. This concern can be addressed in part through rigorous quality control using both biophysical methods and functional assays. Besides, for some detection systems that only express extracellular domains or specific molecular fragments for detection, the missing domains may also affect the interaction properties, especially for the multiple-pass transmembrane proteins. In addition to the experimental part of large-scale PPI screening, the analysis pipeline also introduces some deviations that need to be standardized in the future. Almost all analyses require the selection of a threshold for the detection of true signal, which is typically selected to maximize the system’s precision or sensitivity. However, the precision and sensitivity of a system need to be evaluated using previously identified positive and negative binding pairs. Previously identified interaction pairs are not always rigorously validated, and defining negative pairs, especially within a limited set of screened proteins, poses a challenge. As a result, the manually selected reference pairs used in analysis can introduce bias and inconsistency. In addition, the measured values are processed in different ways. For example, Shilts *et al*. used the two-way median polish normalization method, while Verschueren *et al*. used the random forest machine learning model to predict the probability of interactions. It is important to use a tailored pipeline for specific systems and optimize the utilization of the statistics data, as data processing can also affect the detection performance.

In addition, the different lower-throughput approaches used for further validation may also lead to different conclusions. For instance, FRET/BRET approaches are sensitive to short-lived transient interactions and can detect PPIs in live cells with location information. However, the results are influenced by the compatibility of fusion proteins with appropriate fluorophores or bioluminescence. SPR is considered by many to be the gold standard in PPI detection because it is highly sensitive and does not require labeling. SPR can monitor interaction dynamics and acquire critical PPI parameters, such as dissociation constant (*K*_d_) and association constant (*K*_a_). However, SPR lacks the cellular context and the results obtained may vary depending on the protein purification methods used. These variations may not reflect the true biological conditions. Therefore, the validation approaches should be tailored to specific cases.

In summary, the accuracy, sensitivity, temporal and spatial resolution of detection techniques, as well as the data analysis and validation approaches, can all affect the results of PPI screenings and cause discrepancies among different systems. These factors need to be integrated and standardized in order to improve the reliability and reproducibility of PPI screening results.

## SUMMARY AND PERSPECTIVE

The successful application of immunotherapy across a wide spectrum of cancer types has demonstrated its tremendous potential as a transformative approach in cancer treatment. This has led to a surge of interest in investigating the communication between tumor and immune cells within the tumor microenvironment, which is mediated by PPIs. However, our understanding of this interaction network is still developing. Only with a comprehensive understanding of the PPI network can we accurately target molecules that are crucial in tumorigenesis, metastasis and relapse. We can also identify highly responsive targets with reduced side effects that are tailored to individual patients. This demand necessitates large-scale screening of PPIs and a panoramic mapping of the protein–protein interactome within the tumor microenvironment that are crucial to regulate immune functions to fight against diseases.

Researchers have improved upon classical experimental methods for studying PPIs to expand the scalability of PPI screenings. They have established various tailored systems, such as ELISA-based systems, cell signaling transduction-based systems and other omics-integrated systems. These experimental methods are also mutually beneficial with computational-based research methods, such as PPI prediction, cell interaction inferring and the establishment of PPI databases. These achievements together contribute to a more comprehensive understanding of the PPI networks.

Despite the advances in experimental and computational methods for studying PPIs, there are still some challenges that need to be addressed. One challenge is the lack of standardization and uniformity among these methods, which can limit the accuracy and sensitivity of large-scale PPI screenings. In addition, there is a lack of integration and cross-validation of PPIs identified in different experimental systems and laboratories. This can make it difficult to interpret results and build a comprehensive understanding of PPI networks. To address these challenges, it is important to develop a series of standardized protocols and procedures for PPI screening and validation. In particular, considering the cellular context and conducting functional validation assays are critical steps for the continued advancement of mapping the protein–protein interactome in the TIME.

It is important to integrate experimental and computational methods to improve the efficiency and accuracy of PPI studies. Experimental methods are generating an increasing volume of PPI data, which continuously enriches existing PPI databases. This is essential for advancing the performance of in silico PPI prediction methods. In silico methods can also expedite the efficiency of experimental screening by preselecting PPIs with high interaction propensity for subsequent experimental screening, which can save valuable resources. Current PPI prediction methods need to be improved to achieve high accuracy and confidence, enabling more precise prediction of interacting interfaces. With the rapid advancement and successful application of artificial intelligence (AI) and deep learning in the biomedical field, we anticipate that novel PPI prediction methods using AI and deep learning will deliver highly accurate and efficient results. More standard and lower-throughput detection approaches should be used to validate target PPIs, which can then provide feedback to in silico prediction methods to improve their performance. Computational methods, including deep learning, should also do a better job of integrating PPI networks and resolving PPIs in a comprehensive view, which would be beneficial for further functional analysis. Overall, experimental and computational methods form a mutually beneficial feedback loop, and should be used together to advance the interactome mapping.

With the continual refinement and establishment of diverse PPI screening systems, it will be possible to map a comprehensive interactome of the immune system. This will allow researchers from diverse fields to conveniently explore various potential interactions that are relevant to their unique research objective. The systematically integrated PPI databases will greatly promote the identification of effective immunotherapy targets and provide a comprehensive and deep understanding of their mechanisms of action. The insights gained from PPI studies will reshape the landscape of cancer treatment in the near future.

## FUNDING

This work was supported by National Key Research and Development Program of China (2022YFA1304500 to M.D.), National Natural Science Fund (HY2021-7, 82270180 and 82293633 to M.D.) and Peking University (BMU2021YJ063, 2023Rencai-1 to M.D.).

## CONFLICT OF INTEREST STATEMENT

M.D. holds the position of Assistant Editor for *Antibody Therapeutics* and is blinded from reviewing or making decisions for the manuscript. R.P. declares no conflicts of interest.

## AUTHOR CONTRIBUTIONS

R.P. wrote and M.D. reviewed and edited the manuscript.

## DATA AVAILABILITY

The authors confirm that the data supporting the findings of this study are available within the article.
